# Concurrent outbreaks of *Escherichia coli* O157:H7 and O157:H39 with high asymptomatic carriage of other Shiga toxin-producing *E. coli* in nursery children, south-east Scotland, United Kingdom, July to October 2022

**DOI:** 10.2807/1560-7917.ES.2025.30.46.2500340

**Published:** 2025-11-20

**Authors:** Amir Kirolos, Marion Muir, Andrew Douglas, Genna Leckenby, Lesley Allison, Graham Mackenzie, on behalf of the Incident Management Team

**Affiliations:** 1 National Health Service, Lothian, Directorate of Public Health and Health Policy, Edinburgh, Scotland, United Kingdom; 2 Environmental Health Department, East Lothian Council, Lothian, Scotland, United Kingdom; 3 Public Health Scotland, Scotland, United Kingdom; 4 Scottish *E. coli* O157/STEC Reference Laboratory, Royal Infirmary of Edinburgh, Scotland, United Kingdom; 5 The members of the team are listed in the Supplement.

**Keywords:** STEC, outbreak, carriage, nursery, E. coli, Shiga toxin, food-borne

## Abstract

We managed a complex outbreak of Shiga toxin-producing *Escherichia coli* (STEC) and *stx*-negative *E. coli* O157 (non-STEC) cases in four nurseries in two neighbouring towns in south-east Scotland, with epidemiological links through shared management, staff or other visits. There were 57 confirmed cases between July and October 2022. Nurseries voluntarily closed to support outbreak control. Subsequent whole genome sequencing identified two separate, unlinked outbreaks of *stx*2a-positive *E. coli* O157:H7 (19 confirmed cases in Nursery 1) and *stx-*negative *E. coli* O157:H39 (17 confirmed cases in Nursery 2). Smaller numbers of six additional STEC and *E. coli* O157 (non-STEC) strains were identified in the four nurseries. Five children from Nursery 1 who tested positive for *stx*2a-positive *E. coli* O157:H7 required hospitalisation, one of whom developed haemolytic uraemic syndrome. Children with other STEC and *E. coli* O157 (non-STEC) strains had few or no symptoms. Overall, five of 19 cases with s*tx*2a subtypes were asymptomatic, compared with seven of nine for *stx*2f subtypes, and 14 of 25 for *stx*-negative subtypes. Given the findings in this setting, further information on the prevalence of asymptomatic STEC carriage by strain, age and geography, and in other contexts, will support understanding and risk management of future outbreaks.

**Key public health message:**

**What did you want to address in this study and why?**

Shiga toxin-producing *Escherichia coli* (STEC) are a group of bacteria that can cause severe illness in young children and can spread easily in nursery/pre-school settings. We managed a complex outbreak in four nurseries in two neighbouring towns in south-east Scotland. We tested all children and staff at the affected nurseries as part of our investigations and set out to study how transmission occurred in this setting.

**What have we learnt from this study?**

Initially, we suspected this was a single outbreak. However, genetic testing of samples (whole genome sequencing) demonstrated that the affected nurseries had separate outbreaks without spread between them. All children requiring hospitalisation were from one nursery and carried STEC with a specific gene (*stx*2a*)*. Children with other types of STEC without this gene in other nurseries had few or no symptoms.

**What are the implications of your findings for public health?**

More information is needed to assess how commonly STEC infection without symptoms occurs in the community and which genes influence this. Our findings informed Scottish guidelines introduced in 2025 that recommend no public health follow-up for *E. coli* without the *stx* gene. Given that not all STEC causes severe symptoms, these guidelines now also recommend against mass testing those without symptoms in similar settings.

## Background

Shiga toxin-producing *Escherichia coli* (STEC) are a group of bacteria which cause gastrointestinal illness. Severe complications including bloody diarrhoea and haemolytic uraemic syndrome (HUS) occur in a proportion of cases, often requiring hospital or intensive care treatment. These STEC strains produce Shiga toxin 1 (ST1) or 2 (ST2) which are encoded by Shiga toxin genes *stx*1 and *stx*2, respectively, and are associated with severe illness [1]. Shiga toxin-producing strains of *E. coli* are most often associated with the O157 serogroup, but other *E. coli* serogroups can also produce Shiga toxins (collectively referred to as non-O157 STEC) and can cause similar clinical outcomes. While Shiga toxin-producing strains are most associated with severe illness, the presence/absence of other genes such as the *eae* gene can also act as an indicator of human pathogenic potential even in the absence of the *stx* gene [1]. Scottish STEC guidance before 2025 recommended that cases carrying *stx*-negative *E. coli* O157 were managed with the same public health follow-up as STEC [2], whereas most other countries focus laboratory and public health efforts on STEC.

Transmission is via the faecal-oral route, and younger children are at higher risk both of severe illness and of spreading STEC infection to others [3]. Outbreaks in nursery settings are therefore considered high-risk and require urgent action.

Scotland conducts enhanced surveillance of STEC through a coordinated national system involving Public Health Scotland (PHS), National Health Service (NHS) health protection teams, and the Scottish *E. coli* O157/STEC Reference Laboratory (SERL). This system integrates laboratory-confirmed case data with outbreak and surveillance information to track infection patterns, guide public health interventions and reduce the impact of STEC-related illness. Scotland had a mean of 144 *E. coli* O157 (including both STEC and non-STEC) and a mean of 105 non-O157 STEC cases annually between 2017 and 2021 (2021 population size: 5,479,900) [4]. The incidence of all *E. coli* O157 (STEC and non-STEC) ranged between 2.1 and 3.0 cases per 100,000 over the period 2017 to 2021 [4]. Based on historic data, increases in cases of STEC often occur in March and between July and September each year. There was a UK-wide STEC outbreak associated with contaminated lettuce in August 2022 [5]. This was found to be unrelated to the outbreak we describe in this report.

## Outbreak detection

A case of STEC (index case subsequently typed as *E. coli* O157:H7; *stx2a*) in a nursery (Nursery 1) was notified to our Health Protection Team in south-east Scotland on 29 July 2022. Our team followed national guidelines to interview and contact trace, and to exclude the case and close contacts from at-risk nursery, school and work settings [2]. A second case (later typed as the same strain) in the same nursery was notified on 1 August 2022. This led to the formation of an Incident Management Team (IMT) which met regularly between 2 August and 13 October 2022 to manage the outbreak, as further cases of STEC and *E. coli* O157 (non-STEC) were identified in this and three other local nurseries with epidemiological links (nurseries with shared management, staffing, or other visits between nurseries). Most other cases were identified in a second nursery (Nursery 2) a week after the initial cases had emerged in Nursery 1, and a smaller number was detected in two additional nurseries (Nurseries 3 and 4, < 5 cases each) later in the outbreak.

This report summarises the epidemiology, management and lessons learnt from this complex outbreak.

## Methods

### Case definitions

Case definitions for Nurseries 1 and 2 applied to any individual identified after 15 July 2022 (2 weeks before the notification of the index case) and were adapted from national guidance [2]. A later date of 8 August 2022 was used for Nurseries 3 and 4.

Symptoms of STEC or *E. coli* O157 (non-STEC) cases included any of: nausea, vomiting, fever, fatigue, abdominal pain or diarrhoea (with or without blood). At the start of the outbreak, we were concerned that there may have been wider exposure, with the initial nursery cases marking the start of a larger outbreak. Accordingly, the case definition (Box 1) included symptoms in the Scottish STEC enhanced surveillance questionnaire [6], but we extended this list to include fatigue which can be observed, for example, with HUS. We considered ‘primary cases’ as children or staff members who attended an affected nursery. We considered ‘secondary cases’ as household or close contacts of primary cases.

Box 1 **Case definitions *Escherichia coli* O157 nursery outbreaks, south-east Scotland, United Kingdom, July–October 2022**

**Possible case**

Any STEC or *E. coli* O157 (non-STEC) symptom^a^ in an individual in the affected NHS board area^b^ in south-east Scotland but no epidemiological link to a child or member of staff at an affected nursery;
Any confirmed STEC or *E. coli* O157 (non-STEC) case in the affected NHS board area in south-east Scotland with no epidemiological link to an affected nursery.

**Probable case**

Any STEC or *E. coli* O157 (non-STEC) symptom in a child or staff member at an affected nursery;
Any STEC or *E. coli* O157 (non-STEC) symptom in a close contact of a child or staff member at an affected nursery.

**Confirmed case**

STEC or *E. coli* O157 (non-STEC) microbiologically confirmed (by culture or PCR) in a child or staff member or in any individual with an epidemiological link to an affected nursery.

NHS: National Health Service; STEC: Shiga toxin-producing *Escherichia coli*.
^a^ Any of nausea, vomiting, fever, fatigue, abdominal pain or diarrhoea (with or without blood).
^b^ An NHS board is a statutory body responsible for the healthcare of a specific population and for delivering frontline NHS services, with population of mainland NHS boards in Scotland ranging from 300,000 to 1.3 million people

### Epidemiological investigations

For all probable and confirmed cases, we completed a national enhanced surveillance form for STEC and *E. coli* O157 (non-STEC) infection. This included data on demographics (including age and sex), clinical symptoms, places visited, recent activities, travel, food consumed and environmental exposures [6]. We recorded dates attended at any nursery, nursery areas accessed, and symptoms during attendance. We interviewed staff to identify epidemiological links with other nurseries in the local area, focused on identifying shared management or staffing or visits between nurseries.

We undertook active case finding for additional cases linked to this outbreak by contacting all household and close contacts of probable and confirmed cases to ask whether they had any STEC or *E. coli* O157 (non-STEC) symptoms. We contacted all staff and children at the four affected nurseries to identify individuals with STEC or *E. coli* O157 (non-STEC) symptoms. All cases of STEC or *E. coli* O157 infection (e.g. a positive *E. coli* O157 slide agglutination result) were routinely notified to the Health Protection Team covering the affected NHS board area at the time of this outbreak. We reviewed those notifications after 15 July 2022 to identify potential links to cases and nurseries in this outbreak. A public health alert was sent out to all general practices in the NHS board area and health protection teams in other NHS board areas of Scotland to increase awareness and encourage testing and reporting of cases with potential links to this outbreak (those visiting the local area or who had close contact with those from the local area).

We calculated an attack rate separately for Nursery 1 and Nursery 2 by dividing the number of confirmed cases in children from that nursery by the total number of children who attended regularly (nursery roll). Because of the small numbers, we calculated attack rate in Nurseries 3 and 4 together, and for staff in all nurseries combined.

### Environmental investigations

Local health protection and environmental health teams and the Scottish Care Inspectorate inspected all affected nursery premises and reviewed food temperature and menu records.

We collected 33 samples from Nursery 1 including swabs (n = 32) of nappy changing areas, kitchen surfaces, a utility room, all nursery rooms, a staff room and toilets, and a water sample (n = 1) from a mop bucket. Swabs were tested for *E. coli* O157, *stx*, total visible counts and Enterobacteriaceae. We reviewed Scottish Water records for Nursery 1. Scottish Water is the publicly owned organisation that provides all public water and wastewater services to households and businesses across Scotland. There were no concerns from Scottish Water records for Nursery 1. Water in Nursery 2 was from the same treatment works as Nursery 1.

The index case lived in a house with garden, neighbouring a field with two sheep. We collected sheep faeces (n = 6 samples) from the field and took three samples of the private water supply (three different sample dates) from the same house. These samples were tested for *stx*, *eae* and *rfb* genes using PCR. Faeces samples from one family pet dog were submitted for testing using immune-magnetic separation for O157 and PCR for *rfb*, *stx1* and *stx2*.

The IMT decided against the need for further environmental samples from Nurseries 2–4.

### Microbiological investigations

Faecal samples from all children and staff who attended affected nurseries, and from those close contacts or household contacts we had excluded from nursery, school or work, were microbiologically tested. Faecal samples were submitted to the NHS Lothian Enteric Laboratory for culture and presumptive isolates of *E. coli* O157 were forwarded to SERL for confirmation and typing. Faecal samples with a negative culture for *E. coli* O157 at the Enteric Laboratory but from patients in high-risk groups were forwarded to SERL for more sensitive testing by PCR in line with Scottish guidance at the time [2]. This ensured that high-risk samples were tested for all STEC and *E. coli* O157 (non-STEC), in addition to those initially testing positive for *E. coli* O157 via culture. Once cases of non-O157 STEC were identified in the outbreak, and wider testing of all children and staff at affected nurseries was undertaken, all outbreak-related faecal samples were sent directly to SERL for PCR testing.

The SERL conducted real-time PCR for the presence of *E. coli* O157 (detection of *rfb*_O157_), and Shiga toxin genes (detection of *stx*1 or *stx*2, including the *stx*2f variant). All *E. coli* O157:H7 isolates were sub-typed using phage typing [7,8]. Whole genome sequencing (WGS) was undertaken when it had been possible to isolate an organism from a PCR-positive sample. Detailed methods of faecal extraction, real-time PCR and WGS are included in the Supplementary material.

We compared the STEC and *E. coli* O157 (non-STEC) strains in this outbreak with historical sequences in the SERL *E. coli* sequencing database (containing sequences from Scottish human, veterinary and food sources) to identify possible links with other contemporaneous and historical Scottish cases. Sequencing files were also sent to the United Kingdom Health Security Agency to enable a comparison with strains detected in England and Wales, and submitted to EpiPulse for comparison with European and other international strains [9].

## Results

### Epidemiology

Box 2 shows the timeline of events in the outbreak. Between 29 July and 13 October 2022, 57 confirmed cases of STEC and *E. coli* O157 (non-STEC) were identified. We identified and followed up a further 75 probable cases, and 479 close/household contacts of probable and confirmed cases. There were only five people meeting the definition for possible cases; they are not considered further in this report.

Box 2 **Timeline of *Escherichia coli* nursery outbreaks, south-east Scotland, United Kingdom, July–October 2022**

**Week commencing 25 July 2022**

- First confirmed case identified 29 July in Nursery 1.

**Week commencing 1 August 2022**

- Second confirmed case identified 1 August in Nursery 1,
- Incident Management Team formed,
- Joint Health Protection and Environmental Health visit to Nursery 1,
- Nursery 1 voluntarily closed alongside provision of advice letters. Children and staff in Nursery 1 excluded under the Public Health etc. (Scotland) Act 2008 [10] and clearance samples requested.

**Week commencing 8 August 2022**

- Environmental sampling in Nursery 1,
- Further confirmed cases identified in Nursery 1,
- Symptomatic children identified in Nursery 2 (identified as having shared management or staffing with Nursery 1),
- Joint Health Protection and Environmental Health visit to Nursery 2,
- Nursery 2 voluntarily closed alongside provision of advice letters.

**Week commencing 15 August 2022**

- Further confirmed cases identified in Nurseries 1 and 2,
- Children and staff in Nursery 2 excluded under the Public Health etc. (Scotland) Act 2008 and clearance samples requested,
- Symptomatic children identified in Nurseries 3 and 4 (Nursery 3 had an epidemiological link to Nursery 2 through a nursery visit),
- Nursery 3 voluntarily closed alongside provision of advice letters,
- Joint Health Protection and Environmental Health visits to Nurseries 1–4.

**Week commencing 22 August 2022**

- Confirmed cases identified in Nurseries 3 and 4 (Nursery 3 shared management and/or staffing with Nursery 4). Children and staff in Nurseries 3 and 4 excluded under the Public Health etc. (Scotland) Act 2008 and clearance samples requested,
- Nursery 4 voluntarily closed alongside provision of advice letters,
- Information and advice helpline opened,
- Nursery 2 reopened.

**Week commencing 29 August 2022**

- Nursery 1 reopened.

**Week commencing 5 September 2022**

- Final confirmed case identified.

**Week commencing 12 September 2022**

- Nurseries 3 and 4 reopened.

**Week commencing 10 October 2022**

- Incident Management Team stood down and incident declared over.

**Figure 1:**
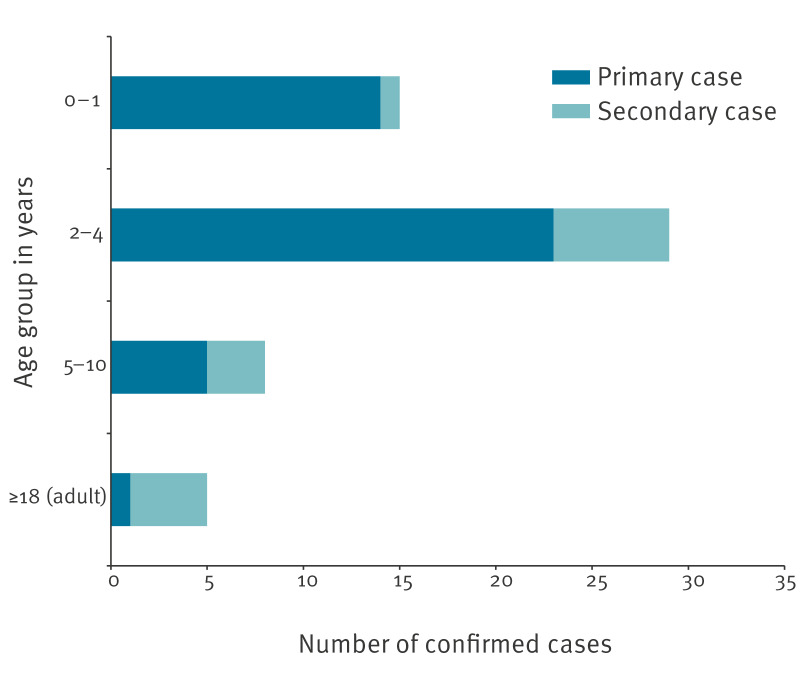
Number of confirmed primary and secondary cases by age group, *Escherichia coli* nursery outbreaks, south-east Scotland, United Kingdom, July–October 2022 (n = 57)

Figure 1 shows the number of confirmed cases by age. Of 57 confirmed cases, 44 were younger than 5 years, and 43 were primary cases (had attended an affected nursery either as child or staff member). The attack rate of STEC and *E. coli* O157 (non-STEC) was 24% for children attending Nursery 1 (27 cases/111 nursery roll) and 12% in Nursery 2 (16/134). Both nurseries were split into three separate age groups, each in their own room. Each nursery had cases spread throughout different nursery rooms and confirmed cases in all three rooms. In contrast, the attack rates for children attending Nurseries 3 or 4 were less than 1%, and the attack rate for staff in all nurseries combined was also less than 1%, with breakdown of numbers not given here to prevent deductive disclosure. There were no staff identified with STEC infection in Nursery 1, removing that as a possible cause for introduction of infection to the nursery.

Primary case: child or staff at affected nursery; secondary case: close/household contact of primary case.

**Figure 2:**
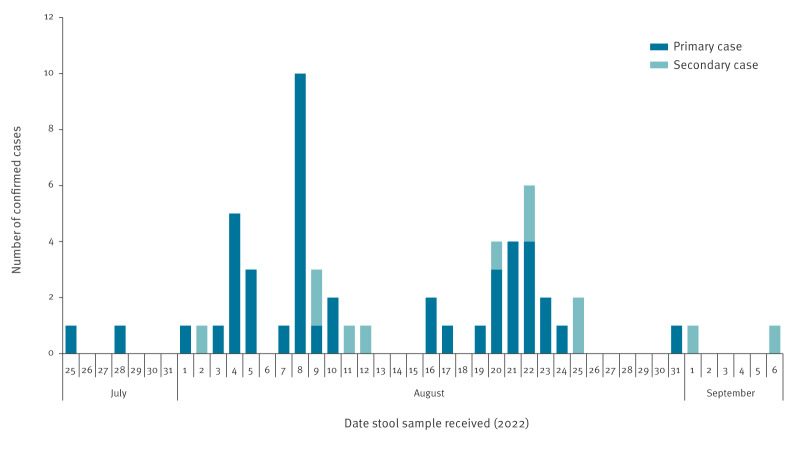
Epidemic curve of confirmed cases, *Escherichia coli* nursery outbreaks, south-east Scotland, United Kingdom, July–October 2022 (n = 57)

Figure 2 shows the epidemic curve of confirmed cases between 25 July to 6 September 2022 based on the date when the laboratory received the sample. This demonstrated an initial peak on 8 August 2022 coinciding with the identification of several cases in Nursery 1, and a second peak on 22 August 2022 that coincided with testing and identification of cases mostly in Nursery 2. Findings and interpretation of results did not change notably when using symptomatology to plot the outbreak curve.

Of the 57 confirmed cases, 28 reported symptoms while 29 were asymptomatic. For the 28 cases reporting symptoms, these included: diarrhoea (n = 21), vomiting (n = 12), fever (n = 11), abdominal pain (n = 6), nausea (n = 4) and bloody diarrhoea (n = 4).

In total, 71 children associated with this outbreak were assessed at the local paediatric hospital, of whom 52 had confirmed STEC or *E. coli* O157 (non-STEC) infection. Five children were admitted to hospital and one developed HUS requiring blood products. There were no deaths.

Enhanced surveillance found no common high-risk foods or environmental exposures between cases.

### Environmental results

Joint environmental health inspections of food temperature/menu logs were satisfactory in all affected nurseries. Nursery 1 was noted on the initial inspection to be, in part, visibly dirty and cluttered. The other three nurseries were noted to be either generally compliant or to have minor actions to address. In total, 33 environmental samples were taken from Nursery 1, three samples were taken from the private water supply of the home of the index case in Nursery 1 on three separate dates and six sheep faeces samples were collected from a field with two sheep neighbouring the index case’s house. No STEC or *E. coli* O157 (non-STEC) was identified in any of these environmental samples.

### Microbiological results

Of 382 individuals tested, 57 cases of STEC or *E. coli* O157 (non-STEC) infection were confirmed. In nine cases, we could not culture an organism (four testing positive for *stx*2 only and five testing positive for *rfb*O157 only); these are reported as ‘not confirmed by culture’. Of the 48 cases confirmed by culture, WGS identified six different *E. coli* serotypes, comprising eight different strains (Table). There were no matches to outbreak strains occurring outside Scotland. 

**Table:** STEC and *Escherichia coli* O157 (non-STEC) serotypes and virulence profile, nursery outbreaks, south-east Scotland, United Kingdom, July–October 2022 (n = 57)

Number of cases	*E. coli* serotype	Virulence profile	Nursery	Phage type^a^	SNP address^b^
19	O157:H7	*eae, stx*2a	1	14/RDNC	18.35.380.738.1009.6697.%
5^c^	O109:H21	*eae, stx*2f	1	ND	9.60.63.66.69.69.%
3	O125:H6	*eae, stx*2f	1	ND	4.4.4.81.93.95.104
3	NCC	*stx2*	1	ND	No SNP address
1	O128:H2	*eae, stx*2f	1	ND	No SNP address
1	NCC	*rfb*	1	ND	No SNP address
1^c^	O157:H16 (A)^d^	*eae*	1	ND	25.25.559.1187.1290.1320.1391
17	O157:H39 (A)^e^	*eae*	2	ND	No SNP address
4	NCC	*rfb*	2	ND	No SNP address
1	O157:H16 (B)^d^	*eae*	2	ND	25.25.249.262.1330.1361.1434
< 5^f^	O157:H39 (B)^e^	*eae*	3	ND	No SNP address
< 5^f^	NCC	*stx2*	4	ND	No SNP address

NCC: not confirmed by culture; RDNC: reactions do not conform (the phage reactions observed did not align with any known profile); SNP: single-nucleotide polymorphism; STEC: Shiga toxin-producing *Escherichia coli*.

^a^ The phage typing scheme is used to type *E. coli* O157:H7. Although sometimes used to type other O157 serogroups, in this case, it did not give any useful typing information so was only used for H7 strains. Only one isolate gave an RDNC result but was a t5 match (fewer than five SNPs differences) with the other strains in this O157:H7 outbreak cluster following whole genome sequencing.

^b^ An SNP address is only produced for certain clonal complexes; % at the end of an SNP address denotes that all strains are a t5 match.

^c^ One case had a dual infection, carrying both *E. coli* O109:H21 and *E. coli* O157:H16 (A).

^d^ The O157:H16 strains fell into two different clusters as determined by different SNP addresses (denoted by (A) and (B)).

^e^ The O157:H39 strains fell into two different clusters following cgMLST analysis using BioNumerics (as denoted by (A) and (B)).

^f^ Specific numbers where there fewer than five cases were involved are not reported to avoid deductive disclosure of individuals.

**Figure 3:**
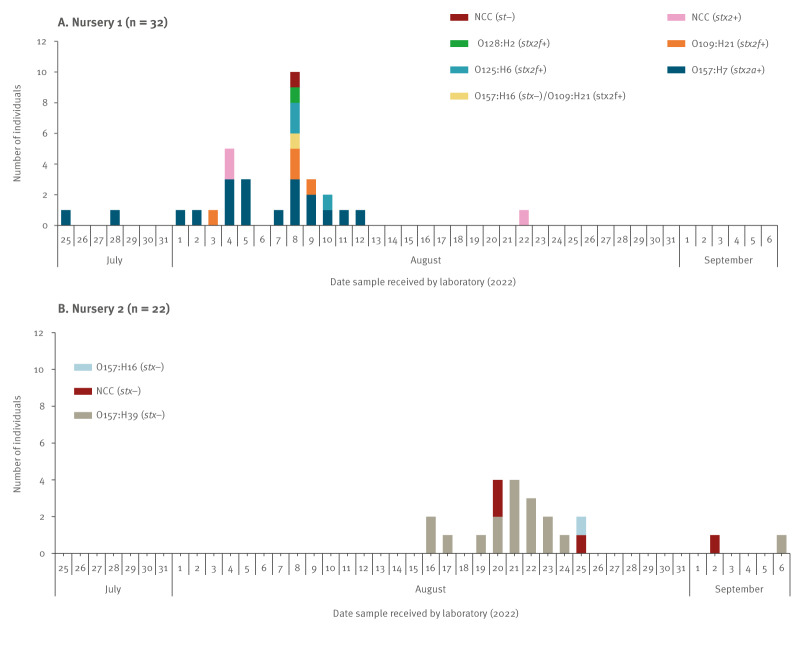
Epidemic curves for Nurseries 1 and 2 by *Escherichia* coli O157/STEC serotype, south-east Scotland, United Kingdom, July–October 2022 (n = 54)

Confirmed cases occurred between 25 July and 22 August in Nursery 1, and between 16 August and 6 September in Nursery 2 (Figure 3). Overall, 19 cases of *E. coli* O157:H7 (*stx2a*) were identified in Nursery 1, and 17 cases of O157:H39 (*stx*-negative) were identified in Nursery 2. Each nursery had distinct clusters as indicated by WGS single-nucleotide polymorphism (SNP) addresses, with no strains common to Nursery 1 and Nursery 2 (Table).

+: positive; −: negative; NCC: not confirmed by culture; STEC: Shiga toxin-producing Escherichia coli.

One case had a dual infection, with both *E. coli* O109:H21 and *E. coli* O157:H16 identified.

Nurseries 3 and 4 each had fewer than five cases of STEC and *E. coli* O157 (non-STEC). We identified *stx*2-positive *E. coli* (not confirmed by culture) in Nursery 3 and *E. coli* O157:H39 (of a different strain to that identified in Nursery 2) in Nursery 4 (Table).

### Symptoms by *stx* subtype

**Figure 4:**
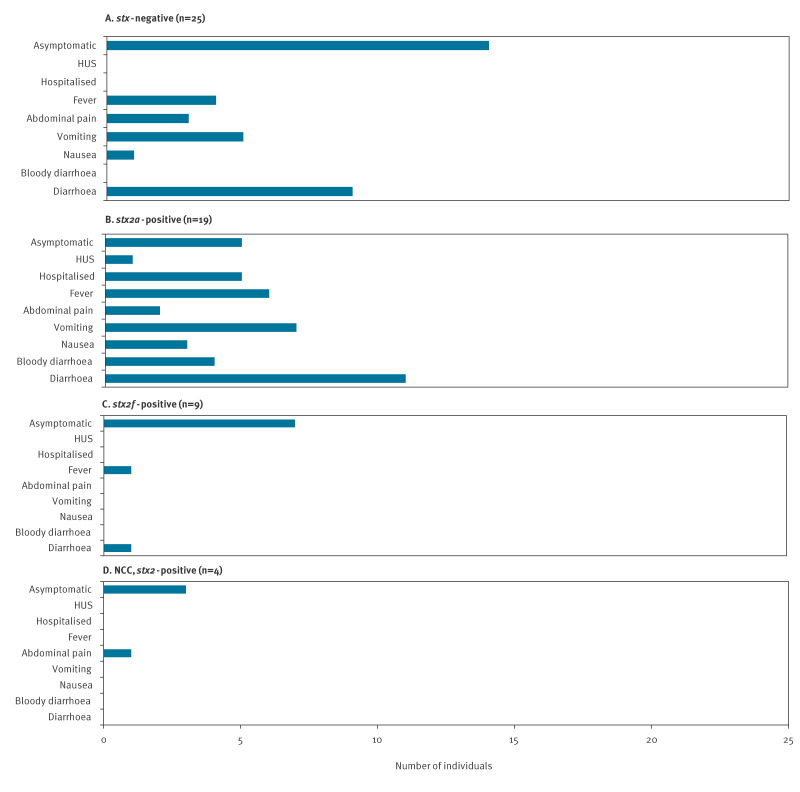
Reported symptoms, hospitalisation, and presence of haemolytic uraemic syndrome by *stx* subtype, *Escherichia coli* nursery outbreaks, south-east Scotland, United Kingdom, July–October 2022 (n = 57)

HUS: haemolytic uraemic syndrome; NCC: not confirmed by culture. Some individuals had more than one symptom.

The only strain to cause severe symptoms in this outbreak *was E. coli* O157:H7 (*stx*2a). Of 19 cases with this *stx* subtype, four had bloody diarrhoea, one of whom developed HUS. Five cases (including three with bloody diarrhoea) were hospitalised (Figure 4). Overall, five of 19 of cases with s*tx*2a subtypes were asymptomatic, compared with 14 of 25 for *stx*-negative isolates, three of four for *stx*2 (not confirmed by culture), and seven of nine for *stx*2f subtypes.

## Outbreak control measures

We contact-traced close/household contacts and interviewed them to identify any STEC or *E. coli* O157 (non-STEC) symptoms, conducting microbiological testing if an individual met the criteria for a probable case. We undertook wide case finding and tested the faeces of all staff and children at the four affected nurseries. Probable and confirmed cases were excluded from nurseries and playgroups under the Public Health etc. (Scotland Act) 2008 [10]. We also excluded household/close contacts from nursery, school or work if they were younger than 10 years (based on uncertain hygiene standards), if their work involved preparing or serving unwrapped foods not subject to further heating, or if they worked as clinical or social care staff who had direct contact with highly susceptible patients [2]. Exclusions from nursery, school or work were applied to all people with a laboratory sample confirmed positive for STEC or *E. coli* O157 (non-STEC) regardless of the presence of symptoms. The subsequent clearance process for confirmed cases and close/household contacts to lift exclusions from nursery, school, or work required two negative cultures for *E. coli* O157:H7 cases, or two PCR negative stool samples for all other strains, 48 h after symptoms had resolved, with the two samples collected at least 24 h apart. When exclusions were put in place, exclusion review meetings were arranged to ensure that no individual was excluded for longer than 3 weeks without regular case review. Exclusions ranged from 20 to 58 days. All confirmed cases had exclusions from nursery, school or work lifted after meeting the clearance requirements. Financial compensation was made available to support compliance of cases and their families with the isolation advice, in line with the guidance for implementing the Public Health etc. (Scotland) Act 2008 [10]. As per the timeline of events (Box 2) Nurseries 1–4 closed voluntarily shortly after cases were identified in their respective settings.

Joint inspection visits were conducted by Environmental Health, Health Protection, the education team from the council area (local authority in Scotland), and the Care Inspectorate (the national agency for regulating and inspecting nurseries and other care services in Scotland). Reports on these inspections were approved by the IMT and shared with the nursery management. Recommendations included decluttering and deep cleaning nurseries, with a particular focus on nappy changing areas and frequently touched contact points. Nurseries were re-inspected before reopening to confirm these actions had been undertaken.

Information and advice letters were sent to parents of children and staff at all four nurseries. The IMT provided media releases and television interviews to provide information to the local community. A National Health Service helpline was set up via NHS24 to answer queries. A clinical pathway with the local paediatrics hospital was developed to allow timely hospital assessment for symptomatic children with links to the outbreak. Confirmed cases were managed through this pathway with supportive treatment for those requiring hospitalisation, and blood products provided to one case with HUS.

No environmental sources for STEC index cases were identified during the outbreak investigations from enhanced surveillance or environmental sampling.

## Discussion

This complex outbreak of STEC and *E. coli* O157 (non-STEC) cases across four nurseries with epidemiological links (shared management, staffing and/or other visits between nurseries) was managed between July and October 2022. Most cases occurred in children from two nurseries, later found to have two concurrent, unrelated outbreaks of *E. coli* O157:H7 (*stx*2a) and O157:H39 (*stx-*negative). Across the four nurseries, wider testing as part of outbreak investigations identified children carrying six additional STEC and *E. coli* O157 (non-STEC) strains with no evidence of transmission between the investigated nurseries. Among all identified strains, *E. coli* O157:H7 (*stx*2a) was the only one associated with severe symptoms. Other strains were linked to mild or no symptoms in most children. We did not find an environmental source for any confirmed cases. However, person-to-person transmission within the nurseries, introduced by index cases for each STEC or *E. coli* O157 (non-STEC) strain, probably caused the concurrent clusters. Several measures including exclusion of cases, contact tracing, exclusion of high-risk close/household contacts, proactive communication, and voluntary nursery closures were used as part of outbreak control. These measures probably contributed to preventing further spread, which was particularly important for the control of *E. coli* O157:H7 (*stx*2a).

We undertook active case finding and performed wide microbiological testing of all staff and children at the four affected nurseries. The outbreak of *E. coli* O157:H39 (*stx-*negative) in Nursery 2 and smaller numbers of children with other strains of STEC and *E. coli* O157 (non-STEC) across all four nurseries were discovered incidentally as a result of this wide testing and would probably not have been reported to local health protection teams otherwise. The 12% attack rate in Nursery 2, where few children reported symptoms, suggests that asymptomatic carriage of *E. coli* O157 (non-STEC) in this group was common. National surveillance data primarily capture symptomatic STEC and *E. coli* O157 (non-STEC) cases. Data to estimate the prevalence of asymptomatic carriage of STEC and *E. coli* O157 (non-STEC) in Scotland by age or location are therefore not readily available. Most confirmed cases in this outbreak were children younger than 5 years, with few cases in other age groups to compare prevalence of asymptomatic carriage by age. The nurseries affected in this outbreak were in a single area in south-east Scotland, with the first two nurseries in a semirural town close to farmland. Children here may have had more and/or a higher risk of environmental and animal exposures than children in more urban settings in Scotland. Given that our testing was carried out in the context of an outbreak among linked nurseries in a semirural community, our findings may not be representative of asymptomatic carriage prevalence at a national level.

Cases and clusters of asymptomatic STEC carriage have been described in other European countries. Young children are higher risk for transmitting STEC [3], although there are limited descriptions of asymptomatic carriage prevalence by age [11,12]. One mixed cross-sectional/prospective study during a national outbreak of STEC O104:H4 in Germany (predominantly affecting adults) looked at 57 cases and 36 control households, and concluded that the prevalence of carriage was low, even in this highly affected setting [13]. A previous study looking at 95 household contacts of patients with HUS found a large proportion of contacts with asymptomatic carriage (18 asymptomatic among 25 STEC-positive) that contributed to secondary transmission; it suggested the protective role of anti-*stx* and anti-O157 lipopolysaccharide antibodies in determining symptoms and severe illness [14]. Transmission and asymptomatic carriage rates in these household settings may be different from the general population. Similarly, close contact and mixing in school and nursery settings may also affect transmission dynamics and rates of asymptomatic carriage compared with the general population [15,16]. Asymptomatic carriers of STEC may pose a lower risk to others than those with symptomatic infection. Sayk et al. looked at asymptomatic long-term carriers of STEC, suggesting that these can be managed using risk-adjusted individual strategies that do not require prolonged restrictions or antibiotic decolonisation [17]. Although strains positive for *stx*2a are most associated with severe illness (as also demonstrated in our outbreak), serogroup cannot be used as a predictor of clinical outcome since all other *stx* subtypes are associated with at least one human case with a severe clinical outcome, and no single or combination of virulence markers is associated exclusively with severe disease [18].

Scottish guidelines introduced in 2025 no longer recommend public health follow-up for *E. coli* O157 (non-STEC), unlike the guidance that was in place during this outbreak [2,19]. Based on the updated guidelines, prompt *stx* typing can be used to guide public health responses and de-escalate actions for *stx-*negative *E. coli* O157 where there is low propensity to cause disease. This aligns with practice and guidelines in other countries that do not require public health follow-up for *stx*-negative *E. coli* O157 [20]. Guidelines and practice in several countries now also recommend different follow-up actions based on *stx* subtype and associated virulence profile [20]. A similar tiered approach to proportionate public health action based on *stx* subtype could be considered for Scotland and other countries based on the emerging evidence. Updated Scottish guidelines now also recommend against the mass testing of asymptomatic individuals in group settings such as nurseries [19]. These recommendations were supported by our outbreak investigation which found limited benefit from this approach given that we incidentally identified several asymptomatic cases and clusters with STEC and *E. coli* O157 (non-STEC) strains unlikely to cause severe illness.

## Conclusion

Our findings suggest that asymptomatic carriage of specific STEC and *E. coli* O157 (non-STEC) strains was common in nursery-aged children in this area of Scotland. Additional information on the prevalence of asymptomatic carriage of STEC strains in the community, particularly by *stx* subtype, age and geography, would be of use to inform future outbreak responses. Learning from this outbreak has already contributed to recent Scottish guidelines and our findings, alongside additional evidence, can inform other countries’ practice in managing STEC.

## Data Availability

The FASTQ reads from sequences in this study have been submitted to the National Centre for Biotechnology Information (NCBI; https://www.ncbi.nlm.nih.gov; BioProject no. PRJNA419720). Strain-specific identifiers can be found in Supplementary Table S1.

## Supplementary Data

155000 Supplement
