# Supplementary material for: Concurrent outbreaks of Escherichia coli O157:H7 and O157:H39 with high asymptomatic carriage of other Shiga toxin-producing E. coli in nursery children, south-east Scotland, United Kingdom, July to October 2022
Source: Euro Surveill. 2025 Nov 20;30(46):2500340. doi: 10.2807/1560-7917.ES.2025.30.46.2500340 (PMC12639274; doi:10.2807/1560-7917.ES.2025.30.46.2500340)
Supplement: Supplement [file 25-00340_MacKENZIE_Supplement.pdf]

Supplement

This supplementary material is hosted by *Eurosurveillance* as supporting information alongside the article "*Concurrent outbreaks of Escherichia coli O157:H7 and O157:H39 with high asymptomatic carriage of other Shiga toxin-producing E. coli in nursery children, south-east Scotland, United Kingdom, July to October 2022*", on behalf of the authors, who remain responsible for the accuracy and appropriateness of the content. The same standards for ethics, copyright, attributions and permissions as for the article apply. Supplements are not edited by *Eurosurveillance* and the journal is not responsible for the maintenance of any links or email addresses provided therein.

E. coli Incident Management Team membership

Please note, on occasion, meetings were attended by junior and trainee staff for training purposes.

Co-Chairs of the IMT

Name	Role	Organisation
Dr Graham Mackenzie	Chair, Consultant in Public Health	NHS Lothian
Josie Murray	Chair, Consultant in Public Health	NHS Lothian
Dr Richard Othieno	Chair, Consultant in Public Health	NHS Lothian

Membership of the IMT

Name	Role	Organisation
Alison Cameron	Quality Improvement for Early Years	East Lothian Council
Amie Borge	Advanced Health Protection Nurse	NHS Lothian
Andrew Douglas	Environmental Health Team Manager	East Lothian Council
Arlene Reynolds	Senior Professional Adviser in Public Health	Scottish Government - observer
Cara Lewis	Infectious Disease	Scottish Government - observer
Carol Calder	Lead Nurse, Edinburgh Infection Control Team	NHS Lothian
Cath Agnew	Care Inspectorate	Care Inspectorate
Cath Morrison	Advanced Health Protection Nurse	NHS Lothian
Chris Tracey	Health Protection Nurse	NHS Lothian

Name	Role	Organisation
Dr Donald Inverarity	Consultant Microbiologist	NHS Lothian
Dr Ewan Olson	Microbiology Consultant	NHS Lothian
Fiona Smith	Business Support Officer/PH HPT	NHS Lothian
Genna Leckenby	Healthcare Scientist Advanced (Epidemiology)	Public Health Scotland
Dr Geoff Foster	Microbiologist, Veterinary Services	Scottish Rural College
Dr Gill Hawkins	Senior Medical Officer	Scottish Government - observer
Holly Macdonald	Environmental Health	East Lothian Council
Jacky Gillan	Service Manager	Care Inspectorate
Jacqueline Dennis	Senior Improvement Advisor	Care Inspectorate
Dr Janine Thoulass	Consultant Public Health Medicine, Clinical Health Protection	Public Health Scotland
Jean Harper	Geographical Lead, Sick Children's hospital	NHS Lothian
Jeni Armstrong	Advanced Health Protection Nurse	NHS Lothian
Jill McKay	Environmental Health Comms Lead	East Lothian Council
Joanne Allen	Communication Lead, Early Years	East Lothian Council
Dr John Cowden	Consultant Epidemiologist	Public Health Scotland
Karen Quinn	Team Manager, Care Inspectorate	Care Inspectorate
Kizzy Taylor	Communications Manager	NHS Lothian
Laura Jones	Consultant Paediatrician	NHS Lothian
Dr Lesley Allison	Principal Scientist/ Deputy Director	Scottish *E. coli* O157/STEC Reference Laboratory (SERL)
Linda Mulhern	Operations Manager, Microbiology Laboratories	NHS Lothian
Lindsay Guthrie	Associate Director, Infection Prevention and Control	NHS Lothian
Louise Wellington	Health Protection Clinical Nurse Manager	NHS Lothian
Lynda Browning	Principal Healthcare Scientist (Epidemiology)	Public Health Scotland
Lynn Crothers	Protective Services Manager, Chief EHO	East Lothian Council
Lynne Ziarelli	Communication Manager	NHS Lothian
Marion Muir	Environmental Health	East Lothian Council
Prof. Matt Holden	Professor of Pathogen Genomics	Public Health Scotland
Nicola McDowell	Head of Education	East Lothian Council

Name	Role	Organisation
Peter Harrison	Nurse Consultant, HPT	NHS Lothian
Dr Pota Kalima	Clinical Lead, *E. coli* ref lab	NHS Lothian
Sharon Saunders	Head of Place	East Lothian Council
Shonagh Szwedowska	Business Support Officer/PH HPT	NHS Lothian
Sinead Donnelly	Senior Communications Office	NHS Lothian
Susan Brownlie	Healthcare Scientist	Public Health Scotland

Extended microbiological methods

Faecal extraction

Each faecal sample was enriched in tryptic soy broth before performing DNA extraction using Instagene (Bio-Rad), prior to polymerase chain reaction (PCR).

Real-time PCR

The Scottish *E. coli* O157/STEC Reference Laboratory (SERL) used an in-house real-time PCR as part of the faecal screening process to detect Shiga toxin genes (*stx*1 and *stx*2), including all common *stx* variants, and a gene specific for *E. coli* O157 (rfb_{O157}). The presence of rfb_{O157} (with or without *stx*) following real-time PCR, indicated the potential presence of *E. coli* O157. The presence of *stx*1 and/or *stx*2, in the absence of rfb_{O157}, indicated the potential presence of non-O157 STEC. The pattern of genes present following real-time PCR determined the methods undertaken to isolate an organism.

Isolation of E. coli from positive PCR faeces

As non-sorbitol fermenting (NSF) *E. coli* O157 possess a unique biochemical characteristic, they can be detected on a special culture media and can be isolated either by direct plating or by immunomagnetic separation. The isolation of non-O157 STEC and sorbitol fermenting (SF) *E. coli* O157 involves carrying out individual PCR reactions on multiple colonies on a culture plate (as these non-O157 STEC and SF *E. coli* O157 look like most other organisms on a culture plate) to identify the PCR positive organism. If an organism cannot be isolated following a positive PCR, no further typing can be performed.

Phage typing

All NSF *E. coli* O157 isolates were sub-typed using phage typing[1,2] which determines the susceptibility of *E. coli* O157:H7 strains to a panel of sixteen different bacteriophages. The resulting pattern was scored against the international phage typing scheme and a phage type assigned.

Whole Genome Sequencing (WGS)

Genomic DNA was manually extracted from each *E. coli* organism under investigation using the DNeasy Blood and Tissue Kit (Qiagen, Crawley, UK). Libraries were prepared using the Nextera XT DNA kit (Illumina, Cambridge, UK) and pair-end sequencing performed on the Illumina MiSeq using 500 cycle v2 reagent kits to produce 2 x 250bp reads. Sequencing files (fastqs) were analysed using the Scottish Microbiology Reference Laboratories WGS Pipeline (SMiRLWBP) and BioNumerics v8 (Applied Maths) using the cgMLST and *E. coli* genotyping plug-in tools.

Outputs from the bioinformatic analysis workflow, used to characterise the strains and determine their genetic relatedness, were predicted serotype (O:H), sequence type (ST), Shiga toxin gene (*stx*) subtype and presence/ absence of *eae* (thought to be an indicator of human pathogenic potential), cgMLST allelic profile, and SNP address.

Single-nucleotide polymorphism (SNP) addresses were produced by sending files (JavaScript Object Notation (JSONs)) generated by the SMiRLWBP pipeline to the UK Health Security Agency (UKHSA) for processing to enable a comparison with organisms circulating in England and Wales and to obtain a UK-wide nomenclature. UKHSA performed hierarchical single linkage clustering on the pairwise SNP difference between all isolates at various distance thresholds (Δ250, Δ100, Δ50, Δ25, Δ10, Δ5, Δ0) to produce a seven-digit SNP address that can be used to describe the population structure. Isolates with identical SNP addresses or with fewer than five SNPs differences (termed a t5 match) are considered closely related and likely to have an epidemiological link. Similarly, cgMLST allelic profiles with a maximum of three loci separating the isolates are investigated as they may have an epidemiological link.

1. Ahmed R, et al. Phage-typing scheme for Escherichia coli O157: H7. *The Journal of Infectious Diseases*. 1987; 155(4): 806-809.

2. Khakhria R, Duck D, and Lior H. Extended phage-typing scheme for Escherichia coli O157: H7. *Epidemiology & Infection*. 1990; 105(3): 511-520.

Supplementary Table S1. Table detailing the NCBI FASTQ SRA accessions for finalised assemblies from cases associated with nurseries in South-East Scotland.

Nursery	Serotype	*stx* subtype	*eae*	NCBI Accession
1	O157:H7	*stx2a*	*eae*	SRR21542022
1	O157:H7	*stx2a*	*eae*	SRR21542020
1	O157:H7	*stx2a*	*eae*	SRR21542023
1	O157:H7	*stx2a*	*eae*	SRR21541999
1	O157:H7	*stx2a*	*eae*	SRR21542000
1	O157:H7	*stx2a*	*eae*	SRR21542001
1	O157:H7	*stx2a*	*eae*	SRR21542002
1	O157:H7	*stx2a*	*eae*	SRR21542003
1	O157:H7	*stx2a*	*eae*	SRR21542011
1	O157:H7	*stx2a*	*eae*	SRR21541998
1	O157:H7	*stx2a*	*eae*	SRR21542012
1	O157:H7	*stx2a*	*eae*	SRR21542013
1	O157:H7	*stx2a*	*eae*	SRR21542014
1	O157:H7	*stx2a*	*eae*	SRR21542016
1	O157:H7	*stx2a*	*eae*	SRR21542018
1	O157:H7	*stx2a*	*eae*	SRR21542019
1	O157:H7	*stx2a*	*eae*	SRR21542007
1	O157:H7	*stx2a*	*eae*	SRR21542009
1	O157:H7	*stx2a*	*eae*	SRR21542004
1	O109:H21	*stx2f*	*eae*	SRR21542021
1	O109:H21	*stx2f*	*eae*	SRR21542015
1	O109:H21	*stx2f*	*eae*	SRR21542017
1	O109:H21	*stx2f*	*eae*	SRR21542006
1	O109:H21	*stx2f*	*eae*	SRR21542010
1	O125:H6	*stx2f*	*eae*	SRR21541997
1	O125:H6	*stx2f*	*eae*	SRR21542008
1	O125:H6	*stx2f*	*eae*	SRR21542005
1	O128:H2	*stx2f*	*eae*	SRR35675858
1	O157:H16	-	*eae*	SRR35675859
2	O157:H39	-	*eae*	SRR35675851
2	O157:H39	-	*eae*	SRR35675846
2	O157:H39	-	*eae*	SRR35675844
2	O157:H39	-	*eae*	SRR35675842
2	O157:H39	-	*eae*	SRR35675843
2	O157:H39	-	*eae*	SRR35675848
2	O157:H39	-	*eae*	SRR35675841
2	O157:H39	-	*eae*	SRR35675847
2	O157:H39	-	*eae*	SRR35675857

2	O157:H39	-	*eae*	SRR35675852
2	O157:H39	-	*eae*	SRR35675853
2	O157:H39	-	*eae*	SRR35675854
2	O157:H39	-	*eae*	SRR35675855
2	O157:H39	-	*eae*	SRR35675856
2	O157:H39	-	*eae*	SRR35675838
2	O157:H39	-	*eae*	SRR35675850
2	O157:H39	-	*eae*	SRR35675845
2	O157:H16	-	*eae*	SRR35675849
3	O157:H39	-	*eae*	SRR35675840
3	O157:H39	-	*eae*	SRR35675839
